# Morphological and Proteomic Analysis Reveal the Role of Pistil under Pollination in *Liriodendron chinense* (Hemsl.) Sarg

**DOI:** 10.1371/journal.pone.0099970

**Published:** 2014-06-12

**Authors:** Ming Li, Kun Wang, Xin Wang, Pingfang Yang

**Affiliations:** 1 Key Laboratory of Plant Germplasm Enhancement and Speciality Agriculture, Wuhan Botanical Garden, Chinese Academy of Sciences, Wuhan, China; 2 University of Chinese Academy of Sciences, Beijing, China; Institute of Botany, Chinese Academy of Sciences, China

## Abstract

Pollination is an important physiological process during which interaction between pollen and pistil occurs. This interaction could determine whether or not fertilization will occur and hence the ratio of plant seed setting. *Liriodendron chinense* (Hemsl.) Sarg. (*L. chinense*) exhibits a distinct phenomenon where seed setting ratio is not more than 10% in natural environment. To explore the origin of this phenomenon, we conducted a comparative morphological and proteomic analysis on *L. chinense* pistils upon pollination. The morphological analysis showed that pollen grows well *in vitro*, but much slower on pistil or nutrient medium containing pistil extract. Proteomic analysis showed that 493 proteins had changed the expression after pollination. Among them, 468 and 51 proteins were identified by isobaric tags for relative and absolute quantitation and two-dimensional gel electrophoresis respectively, and 26 proteins were common in the two methods. After proteins functional categorization, 66 differentially expressed proteins that are involved in reproduction process were found. Further analysis showed that among the reproductive process related proteins, protein disulfide-isomerase A6 and four embryo-defective proteins showed closer relations with the low seed setting phenomenon. The results indicated that the element from pistil might be the main reason leading to low seed setting in *L. chinense*, which will provide new insights in the mechanisms underlying *L. chinense* reproduction process.

## Introduction

*Liriodendron* is a genus in *Magnoliaceae* family. Plants in genus *Liriodendron* are distinctive and produce valuable hardwood with great ecological and economic values. They grow fast and the wood is light and soft, and therefore, are cultivated in many temperate mountains of America and China for wood production [1]–[5]. They are flowering plants with beautiful leaves and are used in urban landscaping as they also provide shading. In addition, *Liriodendron* is valued as source material for honey production, chemical extracts [6]–[8], and biofuels [9], [10].

The genus *Liriodendron* survived from the last Ice Age and was distributed in temperate regions in the northern hemisphere over great geographical ranges [11], [12]. Currently it comprises of only two morphologically similar species, *Liriodendron tulipifera* L. and *Liriodendron chinense* (Hemsl.) Sarg., derived from North American and East Asian respectively [11]. However, *Liriodendron chinense* (Hemsl.) Sarg. (*L. chinense*) has been deemed a rare and endangered species because it occurs in small, insular and sparse populations [12]. In 1992, *L. chinense* was listed in the Red List of Endangered Plants in China [13], and in 1998, it was classified as near-threatened species in IUCN Red List of Threatened Species by the International Union for Conservation of Nature and Natural Resources.

Low seed setting percentage is a marked trait in sexual reproduction of *L. chinense*. After years of studies, the setting percentage of *L. chinense* has been shown to be not more than 10% in natural conditions, and it is hard to find the seedling in natural environments [14]. In the last two decades, numerous researchers have conducted studies, such as examining the relative contribution of pollen fertility and transfer, availability of resources, flower, or seed predation and genetics, to determine why *L. chinense* only produces so few seeds [15]–[18]. Unfortunately, there has been no consistent conclusion. Pollination, which is a key event in reproductive processes of plants, especially in rare or endangered plant species like *L. chinense* that have low seed production, is probably one of the weak links in the reproductive cycle. Any barrier occurring between pollen and stigma interaction will lead to low seed production. However, few studies have focused on pollination in *L. chinense*. Zhou and Fan examined pollen quality, pollen germination and growth on stigma using fluorochroma method. The results indicated that *in vivo* the pollen grains can load on about 64% pistils of the gynoecium, but the rate of pollen tube passing the style is low, only 24% [19]. In addition to few pollen tubes passing the style, the pollen tubes may grow twined or in no direction, suggesting that only a smaller percent of the pollen tubes penetrates the micropyle and enter the ovule [20], [21]. The results showed that interactions between pollen and stigma occur in different phases after pollen grains land on the stigma, and that there are various barriers distributed throughout the stigma surface, style and ovule in course of pollen tube growth.

In self-compatible plants, pollen-stigma interactions comprises of six stages between the pollen and the pistil: pollen capture and adhesion, pollen hydration, pollen germination, penetration, growth of pollen tube through the stigma and style, pollen tube enter into the ovule and discharge the sperm cells [22]. After the pollen-stigma interaction, the nuclei of two gametes fuse to form the zygote. However, in self-incompatible plants, no matter the stage where barriers occur, there is no formation of a viable zygote. Previous studies in *L. chinense* showed that many pollen grains germinated on pistils of the gynoecium but few pollen tubes could penetrate the pistil style, and most of the pollen tube couldn't pass through micropyle and enter into ovule. This phenomenon suggests that there might be other factors affecting pollen-stigma interaction in *L. chinense*. To verify this hypothesis, we conducted a systematic morphological and proteomic analysis on the pistil of *L. chinense* during pollination. The results provide new insights on the mechanism underlying sexual reproduction in *L. chinense*.

## Materials and Methods

### *L. chinense* growth conditions and artificial pollination

The *L. chinense* plants were grown in Wuhan Botanical Garden, Chinese Academy of Sciences. During the flowering season, which extends from late April to May, the branches with flower buds which were about to open were cut from the tree and cultivated with half-strength Hoagland's nutrient solution in greenhouse under 14 h light (400–800 µmol m^−2^ s^−1^) at 26±2°C and 10 h darkness at 20±2°C [23]. The relative humidity was maintained at 60–70% [19]. The flower buds with an opening on top and a probability of opening the following day were chosen and the androecium was emasculated at night before pollination. Artificial pollination was done the next afternoon as follow: Mature pollen grains were harvested from open flowers and then they were smeared on the pistils without androecium using a soft brush. This artificially pollinated pistil was cut from the flower 30 minutes after pollination and stored in liquid nitrogen. Similarly, the pistil after 1 h pollination was harvested, stored in liquid nitrogen. The harvested un-pollinated pistil was stored in liquid nitrogen. All three of these samples were named as S2, S3, and S1 respectively and stored in −80°C freezer. All three treatments (S1, S2, and S3) were repeated five times respectively.

### Paraffin section

Anthers and pistils were fixed in FAA solution containing 5% glacial acetic acid, 5% formaldehyde, 70% ethanol at room temperature for 24 h. After dehydration and infiltration, the samples were embedded in paraffin and cut into 10-µm-thick sections by Rotary Microtome Leica RM2265 (Germany). Then the sections were sealed by neutral balsam and photographed by Olympus-BX51 (Japan).

### Gel-based proteomics in *L. chinense*

#### Protein extraction and 2-DE

Proteins of pistils were extracted as previously described [24], [25]. Briefly, 0.25–0.3 g of pistils were ground in 2 mL pre-cooled homogenization buffer which contains 250 mM sucrose, 20 mM Tris-HCl (pH7.5), 10 mM ethylene glycol tetraacetic acid, 1% Triton X-100, 1 mM dithiothreitol, and 1 mM phenylmethylsulfonyl fluoride. The homogenate was centrifuged at 12000×*g* and 4°C for 30 min. Then the supernatant and its 3 volumes cold acetone were mixed in new tube. After keeping the tube at −20°C at least 2 h, the tube was centrifuged at 12000×*g* and 4°C for 30 min. Then the supernatant was discarded followed by washing the protein precipitate with cold acetone three times. After centrifugation, the protein precipitate was vacuum-dried into a protein powder. After centrifugation and quantification, the immobilized pH gradient strips (17 cm, pH 4–7 linear, Bio-Rad, USA) were loaded with 350 µL sample buffer containing 800 µg sample proteins in focusing tray. The focusing tray was kept at room temperature for 16 h. Isoelectric focusing was performed with the PROTEAN IEF system (Bio-Rad, USA) for a total 80000 V-hr. Then the strips were equilibrated in equilibration buffer I (6 M urea, 0.375 M Tris-HCl pH 8.8, 2% SDS, 20% glycerol, and 130 mM dithiothreitol) for 15 min and equilibration buffer II (6 M urea, 0.375 M Tris-HCl pH 8.8, 2% SDS, 20% glycerol, and 135 mM iodoacetamide) for 15 min sequentially. After equilibration, sodium dodecyl sulfate-polyacrylamide gel electrophoresis (SDS-PAGE) was carried out with 12% acrylamide gels. The 2-DE gels were stained with Coomassie Brilliant Blue (CBB) R-250.

#### Image analysis of 2-DE gels

The 2-DE gels were scanned at 600 DPI resolutions with an EPSON PERFECTIONTM V700 PHOTO scanner (Epson (china) Co., Ltd.). The gel images were analyzed with PDQuest 2-DE Analysis Software (Version 8.0, Bio-Rad, USA). Individual spot volume was normalized by total spot volumes per gel to eliminate experimental variations among different 2-DE gels. The average volume of each spot in different experimental treatment was calculated from three biological replicates. The protein spots expression with more than 1.5-fold change (*P*<0.05) among different experimental treatment were regarded as receivable.

#### Protein identification by MALDI-TOF/TOF-MS

The significant differentially expressed spots were excised from the gel manually, and washed with ultrapure water for 20 minutes. Then the spots decolorizations were performed using 100 µL of 50 mM NH_4_HCO_3_ in 50% v/v acetonitrile (ACN) for 1 h at room temperature. After decolorization the gel spots were dehydrated using 50 µL ACN. After drying the gel spots, the proteins were digested according to the method described before [26]. Gernerally, 25 mM NH_4_HCO_3_ containing 10 pmol trypsin (Promega, USA) was added to the tube and kept at 4°C for 1 h, and then it was kept at 37°C overnight. The peptides were extracted and collected from gel spots using three kinds of solution successively (0.1% TFA/99.9% acetonitrile, 0.1% TFA/99.9% H_2_O, 0.1% TFA/50% acetonitrile/49.9% H_2_O). The peptide solution was collected and concentrated to 10 µL, and then desalted by ZipTip C18 pipette tips (Millipore, USA). After peptide solution desalting, 1 µL of the sample solution was loaded on Anchor Chip Standard (Bruker Daltonics Inc, Germany). After the Anchor Chip drying, the matrix solution (saturated solution ofα-cyano-4-hydroxycinnamic acid in acetonitrile, trifluoroacetic acid 0.1%) was loaded on point corresponding to the location of the sample to a target spot. Through ultrafleXtreme (Bruker Daltonics Inc, Germany) Operation, the PMF data was obtained. The instrument parameters for MS acquisition were list as follows: laser intensity was 20%–26%, reflector detector voltage was 2438 V. Protein identification using MS/MS raw data was performed with flexAnalysis software (Bruker Daltonics Inc, Germany) coupled with Mascot Server software (version 2.4.01) based on the NCBI protein database and SwissPort database of green plants. The searching parameters were set as follows: peptide masses were assumed to be monoisotopic, 100 ppm was used as mass accuracy, a maximum of one missing cleavage site, and modifications which included Carbamidomethy and Oxidation were considered. (The timestamp of NCBI protein database is 2011/11/09, there were 949,856 sequences of Green Plants and 5,512,397,590 redundant total sequences in NCBI database; the timestamp of SwissPort database is SwissPort 57.15, there were 28,783 sequences of Green Plants and 515,203 sequences non-redundant total sequences in SwissPort). The proteins which scores greater than 42 (NCBI) or 26 (SwissPort) were chosen (*P*<0.05).

### Gel-free proteomics in *L. chinense*

#### Protein extraction

The proteins were extracted by phenol extraction and methanol/ammonium acetate precipitation as described previously [27]. Then the protein samples for iTRAQ were resuspended in lysis buffer (7 M urea, 2 M thiourea, 30 mM Tris-HCl, 4% CHAPS and 10 mM DTT) in a minimal volume and protein was quantified using BCA protein assay kit (Pierce, USA).

#### Digestion and iTRAQ labeling

About 100 µg proteins of each sample per tube were prepared. Then they were reduced with 12 mM (final concentration) dithiothreitol for 1 h at 37°C. Subsequently, protein alkylations were performed with 50 mM (final concentration) iodoacetamide for 1 h in dark at room temperature. Then the mixture was transferred to centrifugal units (VN01H02, Sartorius, Germany) and centrifuged at 12,000×*g* for 20 min, and then the filtrate was discarded. After centrifugation, 8 mM urea solution was added into the centrifugal units and centrifuged, this step was repeated twice. After that, 100 µL dilute buffer (50 mM triethylammonium bicarbonate) was added into the centrifugal units and centrifuged. Then 50 µL dilute buffer containing 2 µg modified trypsin (Promega) was added into the centrifugal units at 37°C overnight. After trypsin digestion, the resulting peptides were collected using centrifugation. Then the peptides were labeled with iTRAQ reagents (AB Sciex, USA) according to the manufacturer's instructions. For each sample of three time points (i.e., S1, S2, and S3) was iTRAQ labeled 3 times except S3. (i.e., 113-, 116-, 119- tags for S1, 3 replicates. 114-, 117-, 121- tags for S2, 3 replicates. 115-, 118- tags for S3, 2 replicates.)

#### MS/MS Analysis

Labeled peptides were desalted with C_18_-solid phase extraction method and then they were dissolved in strong cation exchange (SCX) solvent A (10 mM ammonium formate, and 0.1% (v/v) formic acid, 25% acetonitrile). After desalting, the peptides were eluted with a linear gradient of 0–25% solvent B (500 mM ammonium formate, 25% acetonitrile) over 90 min followed by 100% solvent B in 10 min using an Agilent HPLC system 1260 with a polysulfoethylA column (2.1×100 mm, 5 µm, 300 Å; PolyLC, USA). After fractionation, the peptides solution was fractionated into 37 fractions, and these fractions were combined into 12 final fractions. A quadrupole time-of-flight (LTQ Orbitrap XL) MS system (Thermo Fisher Scientific, Germany) interfaced with Eksigentnano-LC AS2 system (Eksigent Technologies, CA) using high energy collision dissociation (HCD) was applied as described previously [28]. Finally, 12 fractions were successively loaded onto an Agilent Zorbax 300SB-C_18_ trap column (0.3 mm id×5 mm length, 5 µm particle size) with a flow rate of 5 µL/min. After the fraction loading, reversed-phase C_18_ chromatographic separation of peptides was executed on a pre-packed BetaBasic C_18_ PicoFrit column (75 µm id×10 cm length, New Objective, MA) at 300 nL/min with the following gradient: 5% solvent B for 5 min as an equilibration status; a linear gradient of 60% solvent B for 90 min; 90% solvent B for 5 min as a washing status; 5% solvent B for 10 min as an equilibration status (solvent A: 0.1% formic acid in 98% water, 2% acetonitrile; solvent B: 0.1% formic acid in 98% acetonitrile, 2% water).

#### Database Search and Quantification

The MS/MS data were processed by a thorough search considering amino acid substitution and biological modification against non-redundant NCBI green plants 20131014.fasta (1,544,439 contigs) applying the Sequest algorithm [29] of Proteome Discoverer.1.4 software (Thermo Fisher Scientific Inc.) and the MASCOT algorithm (version 2.4.01; Matrix Science, USA) [30]. For protein identification, the cut-off values were set as follows: proteins detected with more than 95% total ion score confidence interval and more than 2 peptides were chosen. Then S2/S1 or S3/S1 ratios ≥2 and ≤0.5 are considered significantly up- and down-regulated. Protein function analysis by blast2go software (http://www.blast2go.com/b2ghome) was conducted according to the early literature [31]–[34].

## Results and Discussion

### Microscope observation of gametophytes

**Figure 1:**
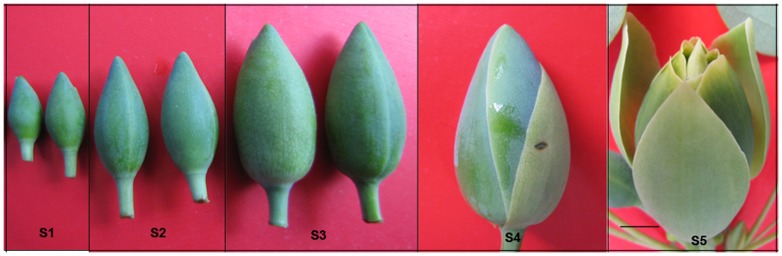
Different development stages of flower buds. S1: the flower buds appear on the branch; S2: ten days after S1; S3: twenty days after S1; S4: the expand period of flower buds; S5: the flower will open soon. Bar in image S5 is 1 cm, and images S1, S2, S3, and S4 have the same magnification as image S5.

**Figure 2:**
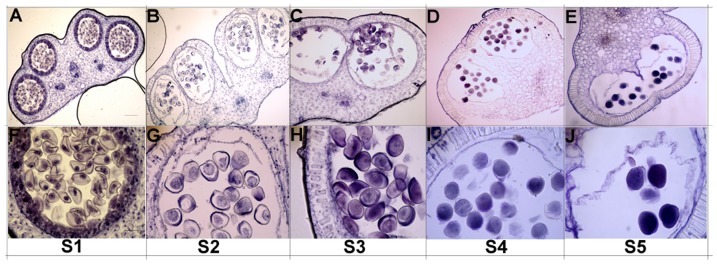
Transverse paraffin section images of anther. Image A and F show the anther in S1 stage; Image B and G show the anther in S2 stage; Image C and H show the anther in S3 stage; Image D and I show the anther in S4 stage; Image E and J show the anther in S5 stage. Bar in image A is 50 µm, and the images B, C, D, and E have the same magnification as image A. Bar in image F is 10 µm, and the images G, H, I, and J have the same magnification as image F.

**Figure 3:**
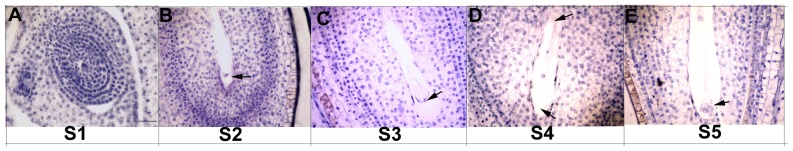
Longitudinal paraffin section images of embryo sac. Arrow in image D shows the abnormal egg cell in embryo sac, arrow in images B, C, and E shows the normal egg cell in embryo sac. Bar in image A is 50 µm, and images B, C, D, and E have the same magnification as image A.

Generally, when the flower matures, the pistil is fully developed and composed of stigma, style, and ovary. Despite different mechanisms of pollination used by different flowers, upon landing on mature stigma, the pollen hydrates and germinates into a pollen tube. During this process, numerous cell-cell interactions occur between the cells of the sporophyte (the pistil) and the male gametophyte (the pollen grain or pollen tube). To explore which one of pollen and pistil might be the major reason that leads to low seed setting ration phenomenon in *L. chinense*, we first observed the development of male and female gametophytes through microscopy. The floral buds were divided into 5 stages according to the diameter, the minimum one with the diameter of approximately 10.0–15.0 mm, and the maximum one with the diameter of approximately 20.0–30.0 mm (Figure 1).Transverse paraffin section images of anther showed that the pollen developed well in different stages (Figure 2). Longitudinal paraffin section images of embryo sac showed that the embryo grew well in early stage, but a few of them grew abnormally at the later stage (Figure 3). These results suggested that the abnormal development of embryo sac in pistil might be part of the reason for low seed setting ratio.

**Figure 4:**
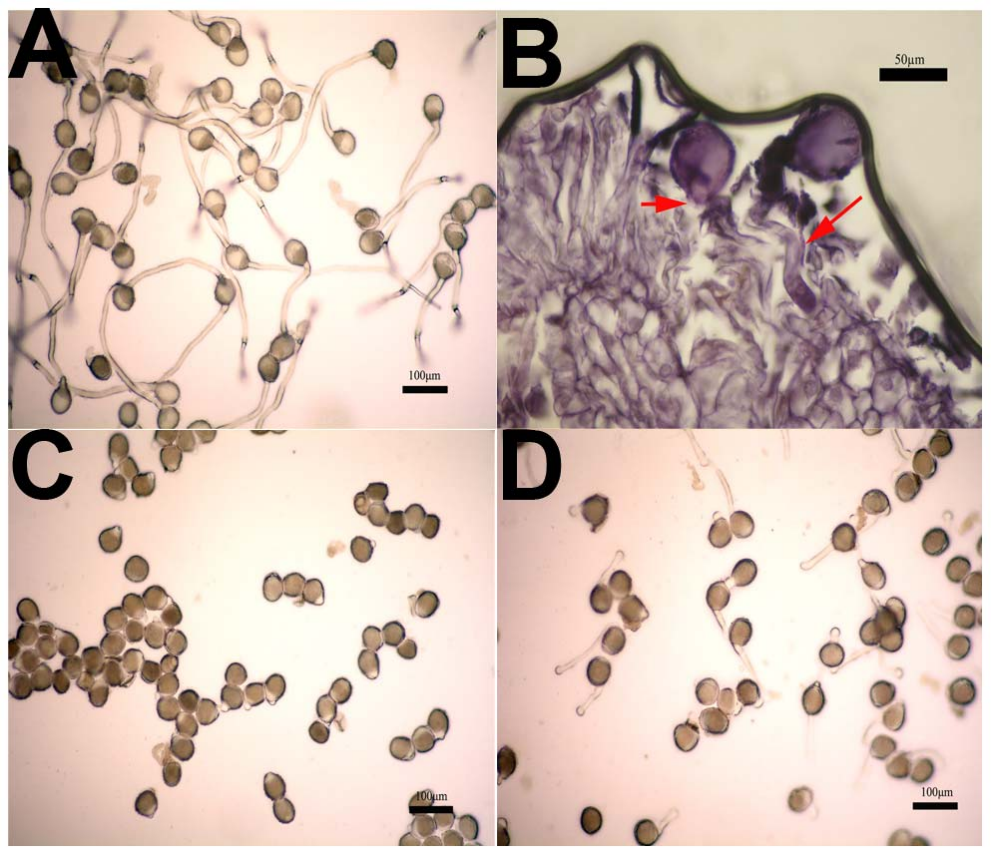
Pollen tubes grow on nutrient medium and pistil. Image A shows pollen tubes grow on nutrient medium after 1; image B shows pollen tubes grow on pistil after 1 h, red arrows in image B indicate the spiral pollen tubes; image C shows pollen tubes almost stop growing on nutrient medium with pistil extract which was added at the beginning of germination; image D shows pollen tubes grew more slowly on nutrient medium with pistil extract which was added after 30 min germination.

To further explore if there are barriers between pollen and pistil interaction, the pollen germination and growth of pollen tube on nutrient medium and pistil were compared. The pollen could germinate normally in both conditions (Figure 4), however, the pollen tube grew differently. It grew well on nutrient medium after 30 min or 1 h (Figure 4A), but very slowly or even stopped on pistil after 1 h (Figure 4B). Moreover, the pollen tube grew in pistil with a malformed status. The pollen tube grew spirally and messily without direction. This observation was consistent with the previous study in inter-specific hybrids (*L. Tulipifera*×*L. Chinense*) [20], [21]. This observation suggested that certain factors from pistil might be the main reason preventing pollen tube growth. To verify this possibility, the pollen grains were germinated and grown in nutrient medium containing pistil extract. The result showed that pollen tube grew more slowly on nutrient medium with pistil extract than on nutrient medium with water (Figure 4C&D). Previous study showed that the pistil extract of Arabidopsis are sufficient to stimulate Arabidopsis pollen germination and induce pollen tube growth [35]. The reasonable explanation of this difference could be that Arabidopsis is self-compatible whereas *L. chinense* have reproduction barrier. Unfortunately, we haven't isolated and identified the effective components from *L. chinense* pistil extract, but all these morphological observations strongly suggested that pistils might be the major factor that results in low seed setting in *L. chinense*.

### Quantitative identifications of pistil proteins using 2-DE and iTRAQ

**Figure 5:**
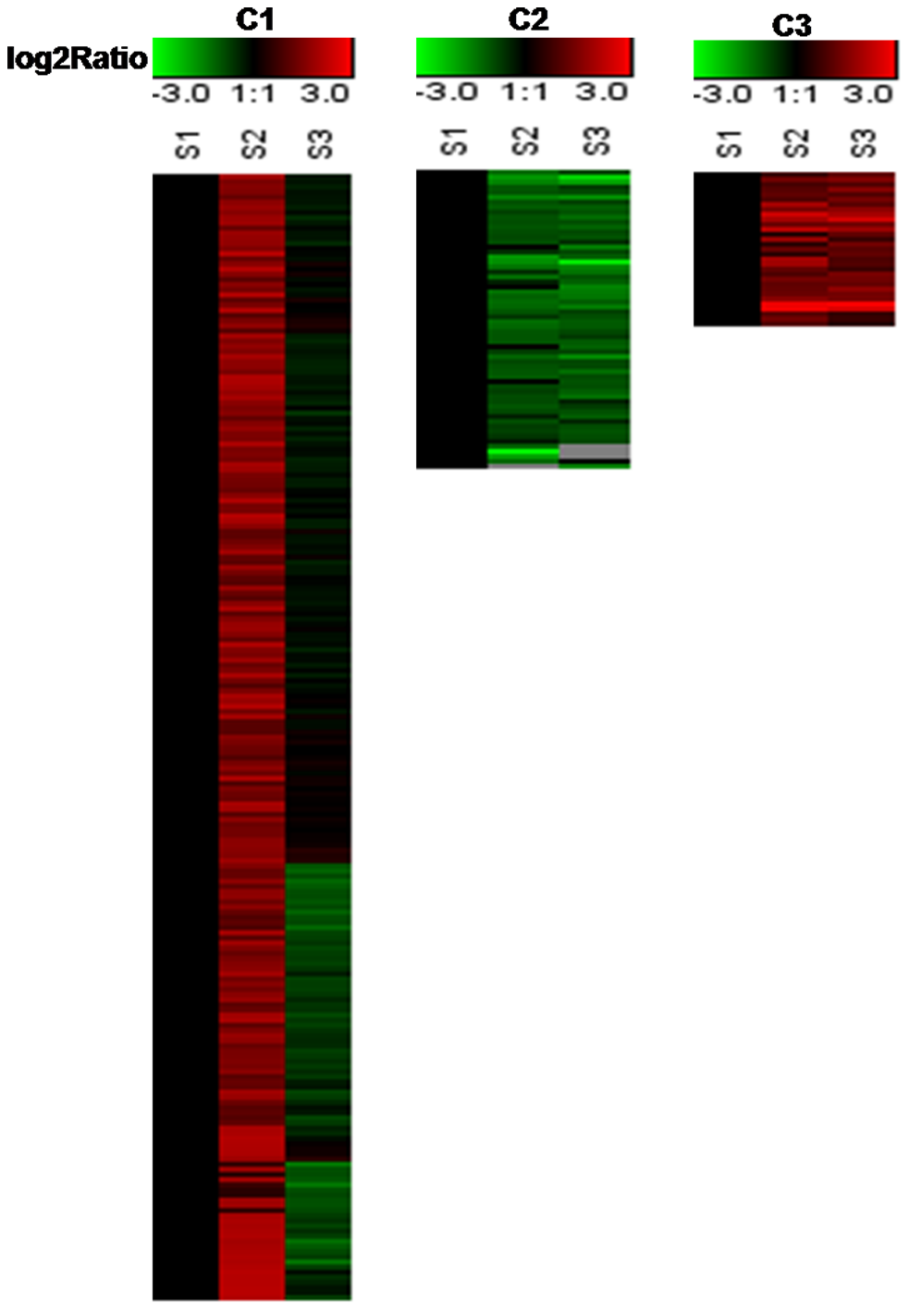
Expression patterns of the differentially expressed proteins. C1: Most of the differentially expressed proteins belong to cluster 1 were up-regulated in S2 and then down-regulated in S3; C2: Differentially expressed proteins belong to the cluster 2 were sustained down-regulated; C3: Differentially expressed proteins belong to the cluster 3 were sustained up-regulated.

To further understand the mechanism underlying this phenomenon, dynamic profiling of the total proteins in *L. chinense* pistils during pollination was conducted using the 2-DE and iTRAQ techniques. Proteins were extracted from pistils of un-pollinated and pollinated flowers with extraction buffer as described in materials and methods. The protein samples were subjected to 2-DE and iTRAQ system. The experimental scheme is shown in Figure S1. Totally, there were 120 protein spots expression changed in 2-DE system (Figure S2) and 468 differentially expressed proteins from iTRAQ system. After merging the data obtained from two techniques, a total of 493 proteins with more than 2-fold-change were identified (Table S1). Among these 493 proteins, 51 proteins were identified from 2-DE system and 468 proteins were identified from iTRAQ system. Further analysis indicated that 26 proteins from 2-DE system were present in iTRAQ system, and these results suggested that there were certain complementary between two methods (Table S1). These differentially expressed proteins have different expressional patterns. In order to show the expressional pattern in more visual way, all the differentially expressed proteins were grouped into 3 distinct clusters according to their expressional patterns by K-means clustering analysis [36]. Most of the differentially expressed proteins belong to cluster 1 which were up-regulated in S2 and then down-regulated in S3. Other differentially expressed proteins belong to the cluster 2 and cluster 3 were sustained down-regulated and up-regulated respectively (Figure 5).

### Functional classification of proteins identified in *Liriodendron chinense* (Hemsl.) Sarg

**Figure 6:**
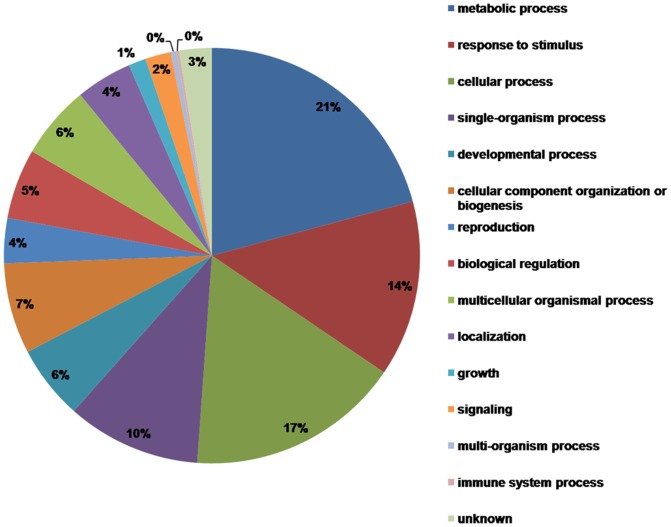
Protein function category.

**Figure 7:**
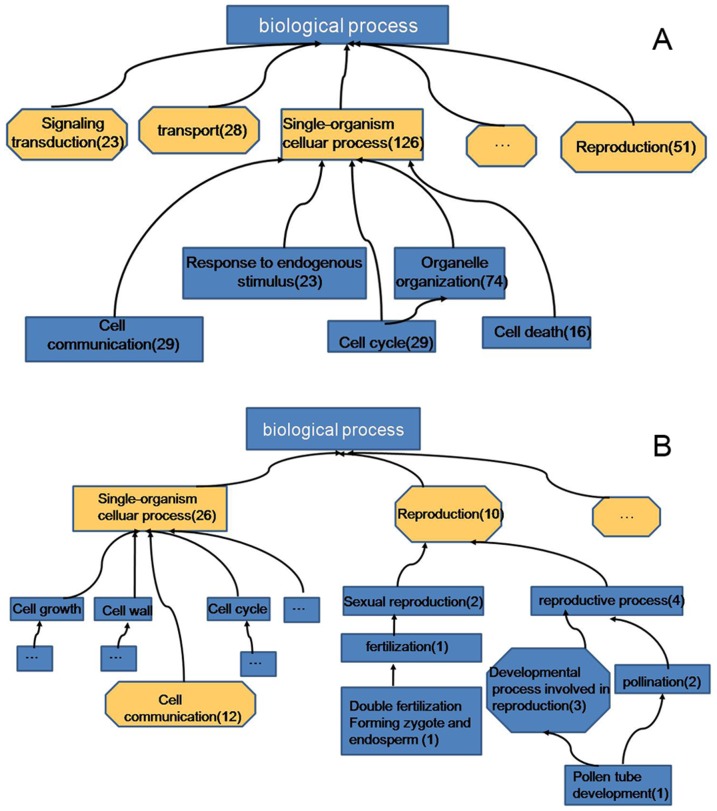
Biological process pathways.

Due to lack of genome information in *L. chinense*, the 493 differentially expressed proteins were blast to the most accurate classification, information in databases (GO annotations, InterPro domains, KEGG), and then the protein functions were analyzed using the blast results by blast2go software(http://www.blast2go.com/b2ghome). Among them, 476 proteins could be sorted into different biological process pathways, which covered a wide range of metabolic and growth pathways (Figure 6). The major functional groups were metabolism related, cellular process related, stimulus response related and single-organism process related. Except these groups, there were also some differentially expressed proteins involved in reproduction, developmental process, localization, growth, signaling and others. Because the biological process pathway result is large and complex, to simplify the data set, the up-regulated proteins and down-regulated proteins were individually characterized and classified according to their putative biological functions by blast2go software (Figure 7).

### Proteins involved in metabolism process and their down-regulated expression suggested the effect of metabolism in *L. chinense* pollination

**Figure 8:**
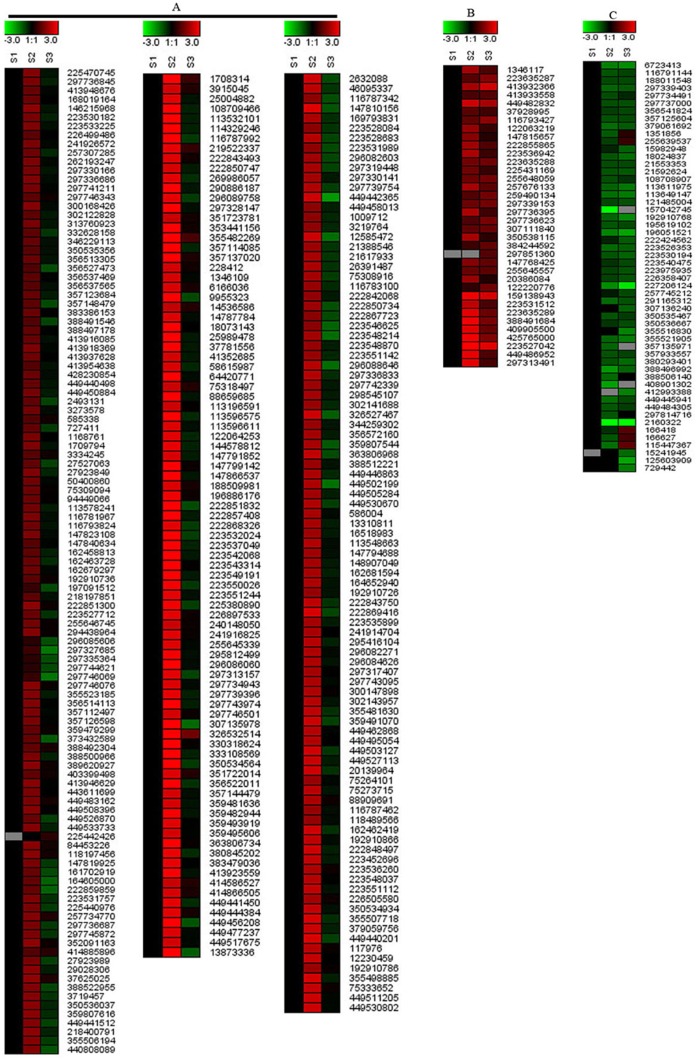
The expression patterns of metabolism related proteins. Cluster A: 295 proteins' expression pattern was up-regulated first and then down-regulated; cluster B: 36 proteins' expression pattern was sustained up-regulated; cluster C: 54 proteins' expression pattern was sustained down-regulated.

In this study, 385 metabolism related proteins were detected to be differentially expressed upon pollination (Figure 8). Among them, 54 proteins were sustained down-regulated, 36 proteins were sustained up-regulated, and most of them involved in cellular metabolic and primary metabolic processes. Another 295 proteins were up-regulated first and then down-regulated, most of them involved in cellular metabolic process, primary metabolic process, organic substance metabolic process, nitrogen compound metabolic process and single-organism metabolic process. Most of the metabolism related proteins were up-regulated first, which indicated that the primary metabolisms were enhanced to facilitate the pollination or protrusion of pollen tube. This result was consistent with the previous study in soybean [25] and rice (unpublished data), which suggested that enhancement of primary metabolisms in pistil might be a common response to the attachment of pollen grains in plant kingdom. However, soybean is strictly self-pollinated whereas rice is self-compatible, and there are no barriers between pollen and pistil interaction in these two species. In contrast, *L. chinense* has a very low seed setting ratio, and there maybe some barriers affecting its pollen-pistil interaction. Different with that in soybean and rice, many of the up-regulated proteins were down-regulated with the extension of pollination time in *L. chinense*. In our study, it might indicate that the metabolism process will weaken in *L. chinense* pistil by the signal regulating from pollen and pistil interaction.

### Differentially expressed proteins reveal new aspects of pollination during reproduction process in *L. chinense*

There were 66 proteins involved in reproduction process (Table S2), which were enriched in the differentially expressed proteins based on the gene ontology (GO) analysis. According to the function categorization and biological process pathway analysis, there were certain proteins involved in embryo development, pollen tube development, double fertilization forming a zygote and endosperm pathway, which we hypothesized to be more interesting than others. Among them, probable protein disulfide-isomerase A6 (Accession number: 729442) might be involved in embryo development, pollen tube development, double fertilization forming a zygote and other process based on the biological process analysis, and its expressional pattern is down-regulated after pollination in *L. chinense*, which suggested that it might be one of the reason for the low seed setting. Although there are few studies focused on disulfide-isomerase A6 in plant, there are many studies on disulfide-isomerase [37], [38]. As a protein folding assistant, they can either catalyze disulfide formation and isomerization or act as a chaperone in many processes [39], [40]. Moreover, it was reported that disulfide-isomerases is involved in double fertilization, embryo sac development and other processes in Arabidopsis. Fertilization was observed to be defective through delaying embryo sac maturation or disrupting pollen tube guidance in disulfide-isomerases mutants [41], [42]. In our study, the expression pattern of disulfide-isomerase A6 was remarkably down-regulated, and the effects of its down-regulation might be similar with the disulfide-isomerases mutants in Arabidopsis. This result strongly suggested the relationship between disulfide-isomerase A6 down-regulation in pistil and the low seed setting ratio in *L. chinense*.

About other four proteins (297339403, 255646745, 297746076, and 414885896), they belong to the EMBRYO-DEFECTIVE (EMB) protein family. Protein 297339403 was sustained down-regulated after pollination, though the others were up-regulated at S2 stage but all of them were down-regulated at S3 stage. The biological process in which these four proteins are involved in and their function were highly relevant to the phenomenon of reproduction in *L. chinense*. In the SeedGenes database (www.seedgenes.org), certain information showed that there were more than 400 genes required for embryo development in Arabidopsis, and many embryo-defective genes were studied through T-DNA mutants [43]–[47]. A larger number of these *EMB* genes encode proteins with an essential function required throughout the life cycle. Recently, a dataset of genes required for gametophyte development in Arabidopsis was established based on information extracted from the literature [44]. In their study, they highlighted 70 pre-globular embryo mutants with a zygotic pattern of inheritance, which provides valuable insights into the maternal-to-zygotic transition in Arabidopsis and the timing of paternal gene activation during embryo development. Besides, they found there were 173 Arabidopsis *EMB* genes with gametophyte-defective phenotype. The EMB protein 297746076 from our results belongs to the 173 Arabidopsis proteins, and one phenotype of proteins 297746076 in Arabidopsis loss-of-function mutation was low seed ratio in siliques of heterozygotes. The other three *EMB* genes (proteins 255646745, 297746076, and 414885896 in our study) belong to 352 true *EMB* genes without evidence of gametophyte defects in Muralla's study. Under the comprehensive analysis of the *EMB* genes related literatures, we propose that there might be more *EMB* genes or proteins in *L. chinense*, and these *EMB* genes might closely link with the low seed setting ratio in *L. chinense*.

## Conclusion

Pollination is a very complex physiological process during which the interaction between pollen and pistil happens. This interaction can determine whether fertilization will occur or not and hence the yield of plant seed setting ratio. *L. chinense* has a distinct phenomenon that the seed setting ratio was not more than 10% under natural condition. To explore the reason why *L. chinense* has such a low seed setting ratio, we conducted a comparative morphological and proteomic analysis on *L. chinense* pistils before and after pollination. The result of morphological analysis showed that the pollen grew well *in vitro*, in contrast to the slow growth in pistil or nutrient medium containing the pistil extract. This observation indicated that the elements from pistil might play major role in *L. chinense* low seed setting ratio phenomenon. Therefore, we investigated the proteins expression in pistil upon pollination. A total of 493 proteins with more than 2-fold changes were identified, and proteins categorization showed that there were 66 proteins involved in reproduction. In addition, disulfide-isomerase A6 and four EMB proteins showed more close relations with the low seed setting ratio. We postulate that these genes might play key role in *L. chinense* reproduction process.

## Supporting Information

Figure S1 **The proteomics experimental scheme.**
(TIF)

Figure S2 **2-DE maps show the protein profile of pistil.** Image A, B and C show the protein profile of pistil in stage S1, S2, and S3 respectively. The black arrows in image A indicate the protein spots which were down-regulated in S2 (S1 *vs* S2); the red arrows in image B indicate the protein spots which were up-regulated in S2 (S1 *vs* S2), the black arrows in image B indicate the protein spots which were down-regulated in S3 (S2 *vs* S3); the black arrows in image C indicate the protein spots which were up-regulated in S3 (S2 *vs* S3).
(TIF)

Table S1 **Total differentially expressed proteins after pollination.**
(XLSX)

Table S2 **Proteins involve in reproduction process.**
(XLSX)
