# Supplementary figures and images for: Morphological and Proteomic Analysis Reveal the Role of Pistil under Pollination in Liriodendron chinense (Hemsl.) Sarg
Source: PLoS One. 2014 Jun 12;9(6):e99970. doi: 10.1371/journal.pone.0099970 (PMC4055720; doi:10.1371/journal.pone.0099970)

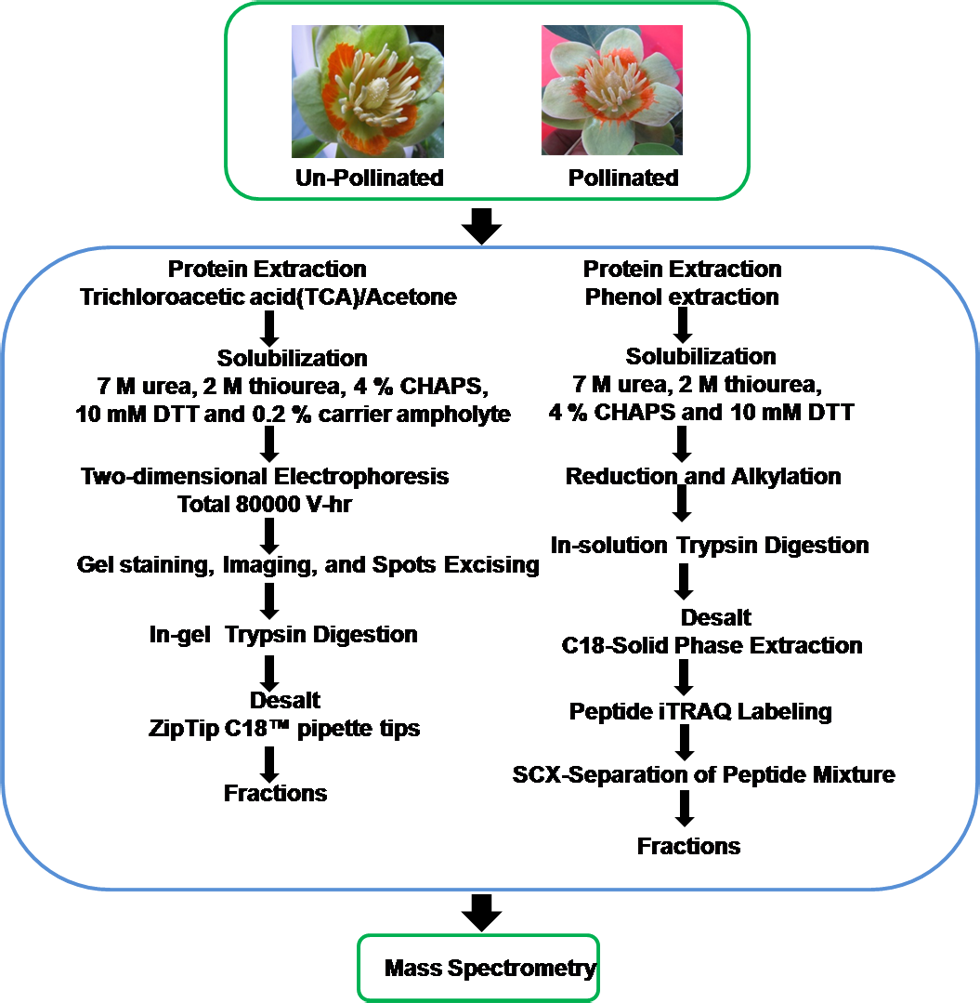

Supplement: Figure S1 — The proteomics experimental scheme. (TIF) [file pone.0099970.s001.tif]

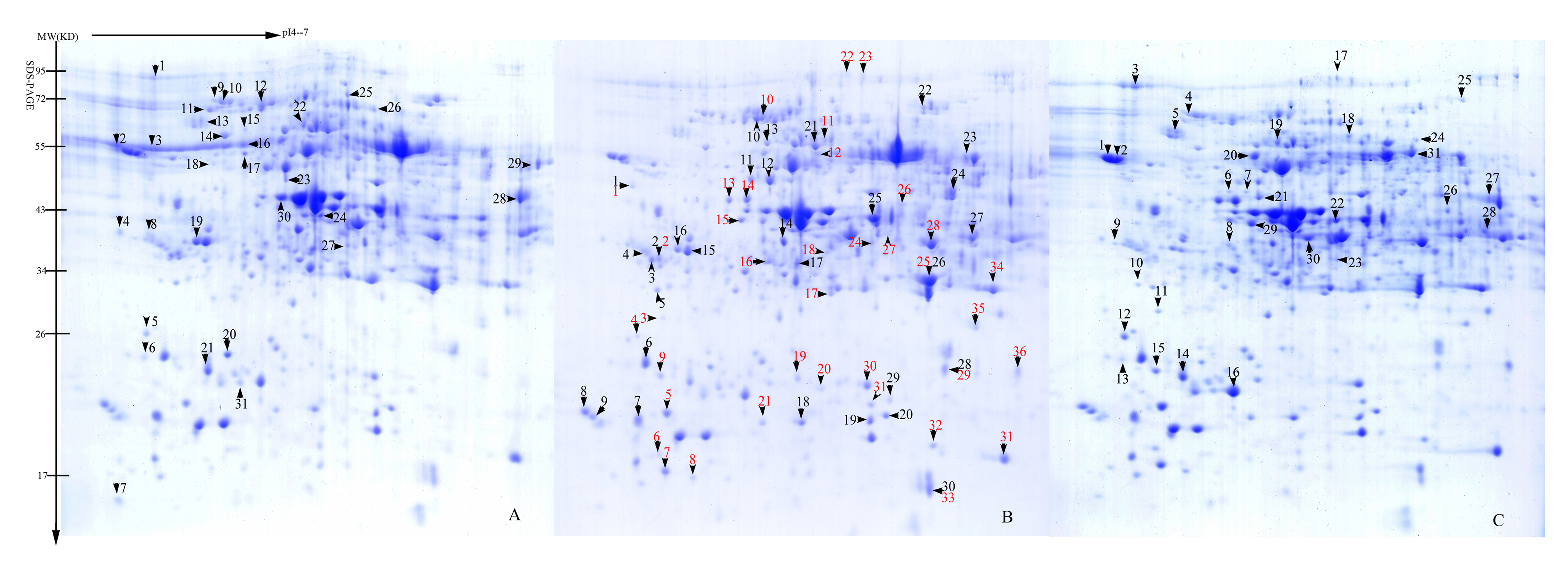

Supplement: Figure S2 — 2-DE maps show the protein profile of pistil. Image A, B and C show the protein profile of pistil in stage S1, S2, and S3 respectively. The black arrows in image A indicate the protein spots which were down-regulated in S2 (S1 vs S2); the red arrows in image B indicate the protein spots which were up-regulated in S2 (S1 vs S2), the black arrows in image B indicate the protein spots which were down-regulated in S3 (S2 vs S3); the black arrows in image C indicate the protein spots which were up-regulated in S3 (S2 vs S3). (TIF) [file pone.0099970.s002.tif]
